# Caregiver Burden in an Online Sample of Family Caregivers: Cross-Sectional Associations with Anticipatory Grief and Family Needs

**DOI:** 10.3390/jcm15208011

**Published:** 2026-10-15

**Authors:** Ana Ramos, Gisele Reami, Ana Querido, Maria dos Anjos Dixe, Carlos Laranjeira

**Affiliations:** 1 School of Health Sciences, University of Leiria and Oeste, Rua General Norton de Matos, 2411-901 Leiria, Portugalana.querido@ulo.pt (A.Q.); maria.dixe@ulo.pt (M.d.A.D.); 2 Centre for Innovative Care and Health Technology (ciTechCare), University of Leiria and Oeste, Campus 5, Rua das Olhalvas, 2414-016 Leiria, Portugal; 3 RISE Health Research Group, University of Porto, 4200-450 Porto, Portugal; 4 Comprehensive Health Research Centre (CHRC), University of Évora, 7000-801 Évora, Portugal

**Keywords:** family caregivers, caregiver burden, anticipatory grief, palliative care, home care, family needs

## Abstract

**Background/Objectives**: Caregiver burden is common among family caregivers providing home-based care and may be associated with anticipatory grief (i.e., the emotional response to an expected loss before death), family-needs satisfaction, and care-related factors. This study aimed to assess the proportion of caregivers reporting burden and examine the independent and incremental associations of burden with characteristics of the caregiver, family, and care recipient in a Portuguese sample. **Methods**: An online cross-sectional, observational, descriptive-correlational study was conducted with 401 family caregivers of individuals with perceived PC needs in Portugal. Family caregivers were recruited through convenience and snowball sampling using healthcare networks, caregiver organizations, and social media platforms. Data were collected through an e-survey comprising sociodemographic and clinical variables, the Zarit Burden Interview (ZBI), the Family Inventory of Needs (FIN), and the Marwit-Meuser Caregiver Grief Inventory-Short Form (MM-CGI-SF). Descriptive statistics, bivariate analyses, and hierarchical multiple linear regression were performed to identify factors associated with caregiver burden. Missing data were handled using complete-case analysis. The study followed the STROBE and CHERRIES guidelines. **Results**: The mean age of caregivers was 55.93 ± 13.30 years, and most participants were female (81.5%). The mean ZBI total score was 57.91 ± 15.23. Using the predefined threshold (ZBI ≥ 46), 77.1% of respondents met criteria for caregiver burden. This proportion comprised 25.2% with mild burden and 51.9% with severe burden. Caregivers experiencing burden reported significantly higher anticipatory grief, lower satisfaction with family needs, and greater patient symptom burden. Hierarchical multiple linear regression explained 56.4% of the variance in caregiver burden (adjusted R^2^ = 0.541). Psychosocial factors accounted for the largest incremental contribution to explained variance (ΔR^2^ = 0.490, *p* < 0.001). In the fully adjusted model, anticipatory grief showed the strongest independent positive association with caregiver burden (β = 0.622, *p* < 0.001), whereas greater fulfilment of care needs was independently associated with lower burden (β = −0.180, *p* < 0.001). **Conclusions**: Findings should be interpreted as cross-sectional associations within a self-selected online sample. Caregiver burden was common among family caregivers providing home-based care and was associated with higher anticipatory grief and lower family-needs satisfaction. Routine assessment and family-centred support interventions may help address caregivers’ needs in this context.

## 1. Introduction

Population ageing and the increasing prevalence of progressive chronic diseases have contributed to a growing global demand for palliative care (PC). International projections indicate that non-communicable diseases will account for 86.1% of all deaths by 2050 [1]. The need for PC is expected to rise substantially in the coming decades due to increasing longevity, functional dependency, and long-term care needs [2,3]. In this context, PC is an essential component of health systems, aimed at improving quality of life, managing symptoms, and supporting patients and their families throughout the disease trajectory [4]. Despite advances in the integration of PC services, substantial inequalities in access persist, particularly in community and home-based settings, where a considerable proportion of care is provided by family members [5,6,7].

The central role of family caregivers in PC is further reinforced by evidence showing that many individuals with PC needs prefer to remain and receive care at home and, whenever possible, to die in their preferred place of care [8,9,10]. Consequently, family caregivers assume substantial responsibility for the provision and coordination of care, often in healthcare systems that rely heavily on informal caregiving, such as Portugal and other European countries [11,12,13]. However, prolonged caregiving at home is associated with significant physical, emotional, and social demands that may adversely affect caregivers’ health and well-being [14]. These demands generally increase as the illness progresses, requiring greater caregiving intensity, time commitment, and emotional involvement. Despite the growing evidence supporting educational, psychoeducational, and technology-based interventions to enhance caregivers’ skills and mitigate psychological distress [15], the support available to family caregivers within formal healthcare systems remains limited and often insufficient to meet their needs [16].

Caregiver burden is among the most frequently studied consequences of informal caregiving and is commonly conceptualized as a multidimensional construct encompassing both objective components (such as caregiving tasks, time demands, and physical effort) and subjective components (including emotional distress, perceived loss of control, and psychological strain) [17,18]. Previous studies have reported moderate to high levels of burden among family caregivers of individuals with advanced illness, with important consequences for mental health, quality of life, and the risk of depression [19,20]. From a stress-process perspective, caregiver burden arises when caregiving demands exceed caregivers’ perceived resources and coping capacity. Within this framework, emotional and contextual factors may play a particularly important role in shaping caregivers’ experiences of strain.

Family caregivers are particularly vulnerable to anxiety, depression, emotional exhaustion, and anticipatory grief. Often described as the “hidden patients,” caregivers may experience levels of suffering comparable to, or even greater than, those of the care recipient [21]. As the illness progresses, caregivers are confronted with multiple losses, including the gradual decline of the patient’s autonomy, changes in family relationships, and increasing awareness of impending death. These experiences may give rise to anticipatory grief, defined as the emotional, cognitive, and relational responses that occur before an expected loss, in contrast to bereavement, which occurs after death [22,23,24].

In parallel, unmet caregiver needs have emerged as another important concern in PC. Family caregivers frequently report unmet needs related to information, emotional support, involvement in decision-making, and practical assistance with caregiving tasks. Evidence suggests that inadequate fulfilment of these needs is associated with greater psychological distress and higher levels of caregiver burden [25,26]. Together, anticipatory grief and unmet family needs may contribute to the caregivers’ perceptions of strain and are therefore relevant dimensions when examining caregiver burden. Consequently, the systematic assessment of caregiver needs has been recommended as a core component of family-centred PC interventions.

In Portugal, support for family caregivers is provided through the National Network of Integrated Continuous Care (RNCCI) and, more recently, through the Informal Caregiver Statute, which formally recognizes caregivers and establishes access to support measures. Nevertheless, important challenges remain regarding the availability, coordination, and accessibility of community-based support services, and many caregivers continue to report unmet support and training needs [9,27]. Structured communication and community-based support interventions have demonstrated potential to improve caregiving experiences and reduce psychological distress [28].

Despite advances in the integration of PC services, significant geographic and organizational disparities persist in access to community and home-based PC. Consequently, family caregivers often assume extensive responsibilities for the coordination and delivery of care, frequently in the context of limited formal support. These demands may increase their vulnerability to caregiver burden, particularly when accompanied by emotional distress and unmet support needs. Although caregiver burden has been widely studied internationally, few studies have simultaneously examined the associations between caregiver burden, anticipatory grief, family-needs satisfaction, and care-recipient characteristics among Portuguese family caregivers. In particular, the role of psychosocial factors, such as anticipatory grief and the extent to which caregivers’ needs are fulfilled, has received comparatively little attention. This knowledge gap hinders the identification of caregivers at increased risk of burden and limits the development of targeted assessment and intervention strategies. Therefore, the aims of this study were to: (1) assess the proportion of family caregivers reporting caregiver burden in an online sample of Portuguese caregivers of individuals with perceived PC needs receiving home-based care and (2) examine the independent and incremental associations between caregiver burden, anticipatory grief, family-needs satisfaction, and caregiver care recipient characteristics. The findings may facilitate earlier identification of vulnerable caregivers, promote family-centred care, and strengthen the capacity of primary healthcare services to deliver comprehensive and sustainable PC in the community.

## 2. Materials and Methods

### 2.1. Study Design

This cross-sectional online survey was conducted among Portuguese family caregivers recruited through healthcare professionals, caregiver organizations, social media platforms, and participant referral. This methodological design allows the characterization of the study population and the exploration of associations between variables of interest, contributing to the identification of factors associated with caregiver burden among family caregivers in the home care setting, without manipulation of the variables under investigation [29].

The study was conducted and reported in accordance with the STROBE (Strengthening the Reporting of Observational Studies in Epidemiology) guidelines [30] and the CHERRIES (Checklist for Reporting Results of Internet E-Surveys) guidelines for online survey methodology [31] (Supplementary file S1).

### 2.2. Participants and Recruitment

The sample consisted of family caregivers of individuals with PC needs, recruited using a non-probability sampling strategy that combined convenience and snowball sampling. Convenience sampling was initially employed through contacts with healthcare professionals and teams, organizations supporting individuals with chronic illness, caregiver support associations, and through dissemination of the study on digital platforms. Individuals who met the eligibility criteria and expressed willingness to participate were included in the study. Participants were eligible if they: (a) were aged 18 years or older; (b) identified as the primary family caregiver, defined as the person providing regular, ongoing, and unpaid care to a relative with PC needs; (c) had been providing care for at least three months; and (d) possessed sufficient proficiency in Portuguese to understand and complete the data collection instruments. Secondary caregivers (individuals providing occasional or supplementary assistance) and individuals who self-reported cognitive difficulties or demonstrated an inability to understand the study information were excluded.

In addition, snowball sampling was used by asking enrolled participants to share information about the study with other family caregivers (who met the inclusion criteria) within their personal or community networks. This approach facilitated access to potential participants who were less easily identifiable through formal institutional channels, thereby increasing sample diversity and enhancing the portrayal of different caregiving contexts. The combination of these sampling methods enabled the recruitment of caregivers with direct experience in providing care to individuals with PC needs while ensuring participation from a range of geographical regions and home care settings. For the purposes of this study, individuals with PC needs were defined as people experiencing serious health-related suffering associated with advanced, progressive, life-limiting, or chronic complex conditions requiring PC interventions aimed at improving quality of life and symptom management. No formal clinical screening instrument, diagnosis-based criterion, prognosis criterion, or clinician confirmation was used to verify PC eligibility.

The target sample size was determined based on the study’s primary objective of estimating the proportion of caregivers experiencing burden. Assuming a proportion of 50%, a 95% confidence level, and a margin of error of 5%, a minimum sample of 385 participants was required. This calculation identified a pragmatic recruitment target and should not be interpreted as conferring population-level precision because participants were recruited through a non-probability sampling strategy. For the multiple linear regression analysis, the achieved analytical sample (n = 387) exceeded the heuristic criterion of N ≥ 50 + 8 m for testing the overall regression model, which corresponds to a minimum sample size of 202 participants for 19 predictors [32].

A total of 427 individuals initiated the survey. Of these, 401 completed all items of the primary outcome measures (burden and anticipatory grief) and were included in the analysis, corresponding to a completion rate of 93.9%. The remaining 26 responses were excluded due to incomplete data on one or both primary outcome instruments.

### 2.3. Instruments

Data were collected using a structured questionnaire administered online via Google Forms. The questionnaire comprised four sections and included instruments selected for their demonstrated validity and reliability in the Portuguese population, as well as their widespread use in research involving family caregivers and PC settings:

(1) **Sociodemographic and clinical characteristics.** Sociodemographic data were collected from family caregivers and included age, sex, marital status, educational level, employment status, relationship to the care recipient, and cohabitation. The care recipient’s symptom burden was assessed using the Edmonton Symptom Assessment System-revised (ESAS-r) [33], completed by caregivers acting as proxy respondents. Participants were instructed to rate each symptom according to their perception of the care recipient’s condition during the period under consideration. The ESAS-r includes nine symptoms (including pain, fatigue, drowsiness, nausea, appetite loss, dyspnoea, depression, anxiety, and overall well-being) rated on a numerical scale ranging from 0 (absence of symptom) to 10 (worst possible symptom severity). Consequently, all symptom scores should be interpreted as caregiver-proxy assessments rather than direct patient reports. This instrument reveals good internal consistency, with a reported Cronbach’s alpha of 0.842. Functional status was assessed using the Portuguese version of the Palliative Performance Scale (PPS) [34]. The PPS evaluates functional performance across five domains: ambulation, activity level, self-care, oral intake, and level of consciousness. Scores range from 0% to 100%, with higher scores indicating better functional status. As data were collected through an online survey, caregivers were asked to report on the five domains of the instrument. PPS scores were subsequently assigned by the research team according to the published scoring guidelines. Consistent with the observational nature of the PPS, caregivers acted as proxy respondents, providing information on the care recipient’s functional status and observable abilities when direct assessment was not feasible [35]. This approach should be regarded as a pragmatic proxy operationalisation rather than a formally validated substitute for professional PPS assessment.
(2) **Family needs.** Family needs were assessed using the Portuguese version of the Family Inventory of Needs (FIN), validated by Areia, Major, and Relvas [36]. The instrument comprises 20 items organized into two subscales: Importance of Care Needs, rated on a 5-point Likert scale ranging from 1 (not important at all) to 5 (extremely important), and Fulfilment of Care Needs, rated on a 3-point scale ranging from 1 (not satisfied) to 3 (satisfied). In the present study, only the total scores of each subscale were used; no item-level analyses were performed. Both FIN subscales exhibited excellent internal consistency, with Cronbach’s alpha coefficients exceeding 0.90 (Importance of Needs: α = 0.95; Satisfaction of Needs: α = 0.959).
(3) **Anticipatory grief.** Anticipatory grief was assessed using the Marwit-Meuser Caregiver Grief Inventory-Short Form (MM-CGI-SF), validated for the Portuguese population by Areia, Major, and Relvas [37]. This instrument consists of 18 items rated on a 5-point Likert scale and is designed to measure anticipatory grief among family caregivers of individuals with an advanced illness. The Portuguese version encompasses three dimensions: Personal Sacrifice and Burden, which reflects the losses and restrictions associated with the caregiving role; Heartfelt Sadness and Longing, which assesses emotional reactions to the progressive deterioration of the care recipient; and Worry and Felt Isolation, which captures concerns about disease progression and social isolation. Higher scores indicate greater levels of anticipatory grief. In this study, the total MM-CGI-SF score was used in the analyses. In the present study, the global scale had a Cronbach’s alpha of 0.902.
(4) **Caregiver burden.** Caregiver burden was assessed using the Portuguese version of the Zarit Burden Interview (ZBI), validated by Sequeira [38]. The instrument comprises 22 items rated on a 5-point Likert scale ranging from 1 (never) to 5 (nearly always), yielding a total score between 22 and 110 points. The Portuguese version includes four dimensions: impact of caregiving, interpersonal relationship, expectations regarding caregiving, and perceived self-efficacy. No items require reverse scoring. Higher scores indicate greater caregiver burden. Caregiver burden categories were established according to the thresholds proposed in the Portuguese validation study: no burden (≤45), mild burden (between 46–56), and severe burden (≥57). In this study, the scale showed a Cronbach’s alpha of 0.899.

### 2.4. Data Collection

Data were collected between July 2025 and April 2026 using an online structured questionnaire administered through Google Forms. The use of this platform enabled efficient survey distribution and data management while providing a cost-effective means of reaching participants across multiple geographic regions and device types [31]. The survey link was disseminated through healthcare professionals and teams, organizations supporting individuals with chronic illness, caregiver support associations, and social media platforms. In addition, participants were encouraged to share the study invitation with other family caregivers who met the eligibility criteria, in accordance with the snowball sampling strategy. Before accessing the questionnaire, potential participants were presented with an information sheet outlining the study aims, procedures, voluntary nature of participation, and data confidentiality measures. Electronic informed consent was obtained prior to participation, and only individuals who provided consent were able to proceed with the survey.

Participants were instructed to complete the questionnaire with reference to the individual with PC needs for whom they were primarily responsible. Information regarding caregiving responsibilities for multiple care recipients was not collected.

To minimise duplicate responses, participants were instructed to complete the survey only once. This information was provided in both the participant information sheet and the survey landing page. The questionnaire required approximately 12–15 min to complete. Data were collected anonymously and stored in a secure database accessible only to the research team. Because the survey was anonymous, no technical methods (e.g., cookies, IP tracking, registration systems) for duplicate-response prevention were available. Upon closure of the survey, the dataset was screened for completeness and eligibility before statistical analysis. No incentives were provided for participation.

### 2.5. Data Analysis

Data were analysed using IBM SPSS Statistics version 31.0 (IBM Corp., Armonk, NY, USA). Descriptive statistics were used to characterize the sample, including frequencies and percentages for categorical variables and means and standard deviations (SD) for continuous variables. Caregiver burden was assessed using the Zarit Burden Interview (ZBI). For descriptive and bivariate analyses, participants were categorized as without burden (ZBI ≤ 45) or with burden (ZBI ≥ 46). For multivariable analysis, the continuous ZBI total score was used as the dependent variable to preserve the full variability of caregiver burden. Bivariate analyses were conducted to compare sociodemographic, caregiving-related, psychosocial, and clinical characteristics between caregivers with and without burden. Categorical variables were compared using Pearson’s chi-square test or Fisher’s exact test, when appropriate. Continuous variables were compared using independent-samples *t*-tests. Normality was assessed using the Kolmogorov–Smirnov test. The findings indicated that only anticipatory grief met the assumption of normality (*p* = 0.200). Although some variables showed departures from normality based on Kolmogorov–Smirnov tests, inspection of distributions and the large sample size supported the use of parametric analyses. Homogeneity of variance was assessed using Levene’s test for independent-samples comparisons. When homogeneity of variance was not met, Welch’s *t*-test was applied. For regression analyses, assumptions were evaluated through inspection of residual diagnostics, including residual plots and normal probability plots [39]. Effect sizes for continuous variables were estimated using Cohen’s d [40]. A hierarchical multiple linear regression was performed to examine variation in caregiver burden. For regression analyses, categorical variables were dummy coded. Sex was coded as female (reference) versus male; academic qualifications were dichotomised into higher education versus non-higher education (no qualifications/basic/secondary education) to avoid sparse categories and improve interpretability; professional situation was coded as no professional activity (reference) versus professional activity; degree of kinship was coded as spouse/partner (reference) versus non-spouse; and cohabitation was coded as living in the same residence (reference) versus not living in the same residence.

No formal adjustment for multiple comparisons was applied. Accordingly, the bivariate analyses should be interpreted as exploratory and descriptive, whereas primary inference was based on the multivariable regression analyses. Predictor variables were included cumulatively in three theoretically defined blocks: caregiver sociodemographic and caregiving characteristics, psychosocial factors, and care-recipient clinical characteristics. Model performance was assessed using R^2^, adjusted R^2^, ΔR^2^, and F-change tests. Regression coefficients (B, β, 95% CI), multicollinearity (tolerance and VIF), residual independence (Durbin-Watson), and influential observations (standardized and studentized residuals and Cook’s distance) were assessed [41,42]. Complete-case analysis was used, with all 19 predictors retained in the final model. Regression assumptions were assessed through examination of residual plots, including the standardized residual histogram, the normal probability plot, and the plot of standardized residuals versus standardized predicted values, as well as through assessment of multicollinearity, residual independence, leverage, and influential observations [40]. To evaluate the robustness of the findings, a sensitivity analysis was conducted by repeating the hierarchical regression after excluding the three observations with the greatest potential influence on the model. These observations were identified based on a combination of absolute studentized residuals, leverage values, and Cook’s distance, with cases demonstrating the strongest overall influence across these diagnostic indicators selected for exclusion. The sensitivity analysis was performed to determine whether the main results were unduly affected by influential observations. As the findings remained substantively unchanged, all observations were retained in the primary analysis. Missing data were handled using complete-case analysis. All statistical tests were two-sided, and significance was set at *α* = 0.05.

### 2.6. Ethical Considerations

This study was conducted in accordance with internationally recognized ethical principles for research involving human participants and was approved by the Ethics Committee of the Polytechnic Institute of Leiria (CE/IPLEIRIA/11/2025). Before accessing the online questionnaire, participants received information about the study objectives, procedures, potential risks and benefits, and the voluntary nature of participation. Electronic informed consent was obtained prior to questionnaire completion. Participants were informed that participation or non-participation would not affect any healthcare services received by themselves or the care recipient. At the end of the questionnaire, information regarding available healthcare and caregiver support resources was provided for participants who experienced distress or wished to seek further support. Confidentiality and anonymity were ensured through the collection of non-identifiable data and alphanumeric coding of records. Access to the data was restricted to the research team, and appropriate measures were implemented to ensure data security and protection. Data will be retained for the period required by institutional research-governance policies and subsequently destroyed in accordance with institutional procedures.

## 3. Results

### 3.1. Sample Description of Participants

The characteristics of caregivers and care recipients are presented in Table 1. Caregivers had a mean age of 55.93 years (SD = 13.30). The majority were female (81.5%), married or living in a common-law relationship (62.8%) and had completed basic education (38.4%). Most caregivers were adult children of the patient (37.9%; n = 152), followed by parents (25.7%; n = 103) and other relatives (16.9%; n = 68), whereas spouses represented 19.5% of the sample. More than half were employed (53.1%), and most lived in the same household as the care recipient (63.3%).

The mean MM-CGI-SF score was 56.02 (SD = 14.22). Participants reported high scores on the FIN Importance of Care Needs subscale (M = 93.69, SD = 8.95), indicating that care-related needs were generally considered important. Scores on the FIN Fulfilment of Care Needs subscale were comparatively lower (M = 50.12, SD = 9.58), which may reflect some discrepancy between the perceived importance of these needs and their fulfilment.

Regarding care recipients, functional status was heterogeneous but generally clustered around a moderate level of impairment (PPS: M = 51.50, SD = 15.90). Furthermore, caregiver-proxy assessments suggested a moderate overall symptom burden, with fatigue (M = 5.83, SD = 2.89), depression (M = 5.32, SD = 3.09), and impaired well-being (M = 5.12, SD = 2.63) receiving the highest ratings.

### 3.2. Distribution of Caregiver Burden Levels

The mean ZBI total score was 57.91 ± 15.23. Using the predefined threshold (ZBI ≥ 46), 77.1% of respondents met criteria for caregiver burden (95% CI approximately 72.9–81.2%). This proportion comprised 25.2% with mild burden and 51.9% with severe burden (Table 2).

### 3.3. Bivariate Analysis According to the Presence or Absence of the Caregiver’s Burden

Bivariate associations between caregivers with and without burden are presented in Table 3 and Table 4. Significant differences in caregiver burden were observed according to the degree of kinship, with spouse caregivers reporting higher levels of burden than non-spouse caregivers [χ^2^(1) = 4.280, *p* = 0.039]. No significant differences in caregiver burden were identified across the remaining categorical variables.

The *t*-test of independent samples results showed that the anticipatory grief of caregivers with burden was significantly higher than that of the non-burden group (t(399) = 12.417, *p* < 0.001, Cohen’s d = 1.553) (Table 4). Moreover, caregivers with burden reported significantly lower levels of fulfilment of care needs than those without burden (t(394) = −6.031, *p* < 0.001, Cohen’s d = −0.723). Caregivers reporting burden also reported lower care recipient functional status than caregivers without burden (t(397) = −2.190, *p* = 0.028, Cohen’s d = −0.260). Caregivers classified as burdened reported higher caregiver-proxy ratings of several care recipient symptoms, including fatigue, drowsiness, appetite loss, depression, anxiety, and impaired well-being (*p* < 0.05). No significant differences were found for age, importance of care needs, pain, nausea or dyspnoea (*p* > 0.05).

### 3.4. Factors Associated with Caregiver Burden

To complement the dichotomised analysis and address potential information loss associated with categorising caregiver burden, a hierarchical multiple linear regression analysis was conducted using the total Zarit Burden Interview (ZBI) score as a continuous outcome variable. Predictors were entered sequentially in three theoretically defined blocks (Table 5), consistent with the multidimensional conceptualisation of caregiver burden and recommendations for hierarchical regression modelling.

Complete-case analysis yielded an analytical sample of 387 caregivers, with the same participants retained across all models. The first block included caregiver sociodemographic and caregiving characteristics; the second block added psychosocial variables (anticipatory grief and family needs); and the third block incorporated care-recipient functional status and caregiver-proxy-rated symptom variables.

Model 1, which included caregiver sociodemographic and caregiving characteristics, was statistically significant, F(6,380) = 2.668, *p* = 0.015, explaining 4.0% of the variance in caregiver burden (R^2^ = 0.040; adjusted R^2^ = 0.025). The addition of anticipatory grief and family-needs variables in Model 2 resulted in a substantial improvement in model fit, accounting for an additional 49.0% of the variance (ΔR^2^ = 0.490, ΔF(3.377) = 130.939, *p* < 0.001). Model 2 explained 53.0% of the variance in caregiver burden (R^2^ = 0.530; adjusted R^2^ = 0.519) and was statistically significant, F(9.377) = 47.249, *p* < 0.001. The addition of care-recipient functional status and symptom variables in Model 3 produced a further statistically significant, although comparatively modest, increase in explained variance (ΔR^2^ = 0.034, ΔF(10.367) = 2.853, *p* = 0.002). Although the addition of the clinical block significantly improved explained variance and yielded the lowest AIC, BIC was lowest for Model 2, suggesting that the more parsimonious psychosocial model may provide a preferable balance between model fit and complexity under the stronger BIC penalty (Supplementary Table S1).

The final model was statistically significant, F(19.367) = 24.983, *p* < 0.001, explaining 56.4% of the variance in caregiver burden (R^2^ = 0.564; adjusted R^2^ = 0.541) (Table 6). In the fully adjusted model, higher anticipatory grief scores were independently associated with higher caregiver burden scores (B = 0.667, β = 0.622, *p* < 0.001). Greater fulfilment of family care needs was independently associated with lower caregiver burden (B = −0.287, β = −0.180, *p* < 0.001). Sex (B = −3.040, β = −0.077, *p* = 0.029), academic qualifications (B = −3.600, β = −0.110, *p* = 0.004), caregiver-proxy-rated pain (B = −0.539, β = −0.110, *p* = 0.008), and caregiver-proxy-rated nausea (B = −0.608, β = −0.109, *p* = 0.012) were also significantly associated with caregiver burden. The remaining variables included in the model had no statistically significant association with ZBI scores after adjustment for the other covariates.

Regression diagnostics supported the adequacy of the model. No evidence of problematic multicollinearity was observed, with tolerance values ranging from 0.513 to 0.957 and variance inflation factors ranging from 1.045 to 1.948. The Durbin-Watson statistic was 1.596, indicating no substantial residual autocorrelation. Standardized residuals ranged from −3.100 to 4.404, and studentized residuals ranged from −3.137 to 4.519. Although a small number of observations exhibited relatively large residuals, influence diagnostics indicated limited impact on model estimates, with a maximum Cook’s distance of 0.061. Overall, diagnostic assessments did not identify major violations of regression assumptions and supported the interpretation of the final model (Supplementary Table S1).

A sensitivity analysis excluding the three atypical observations identified through examination of residual and influence diagnostics (n = 384) yielded substantively unchanged results (Supplementary Table S2). The final model remained statistically significant and explained 58.1% of the variance in caregiver burden (adjusted R^2^ = 0.559), compared with 56.4% (adjusted R^2^ = 0.541) in the primary analysis. The incremental contribution of the clinical block also remained statistically significant (ΔR^2^ = 0.036, *p* < 0.001). Anticipatory grief remained the strongest independent correlate of caregiver burden (β = 0.626, *p* < 0.001), while fulfilment of care needs remained inversely associated with burden (β = −0.186, *p* < 0.001). The direction and statistical significance of the principal associations were therefore substantively unchanged, supporting the robustness of the primary findings.

## 4. Discussion

The present study aimed to assess the proportion of caregiver burden among family caregivers of individuals with perceived PC needs receiving home-based care and to identify factors associated with caregiver burden. The findings revealed a high proportion of burden, highlighting its clinical relevance and the complex interplay of emotional, familial, and clinical factors associated with the caregiving experience in PC.

Home-based care relies heavily on the contribution of family caregivers, who assume central responsibilities in the day-to-day management of illness and the continuity of care. While this arrangement enables many patients to remain at home, in accordance with their preferences, it also imposes substantial demands that may compromise caregivers’ physical and psychological well-being, making it essential to identify the factors associated with caregiver burden. In the present study, the observed proportion of caregivers reporting burden (mild and severe) was 77.1%, with 51.9% of caregivers reporting severe burden. These findings highlight the magnitude of this phenomenon in the context of home-based care. International evidence similarly indicates that caregiver burden is highly prevalent among caregivers of individuals with advanced illness, although direct comparisons across studies are limited by the heterogeneity of cut-off values and burden classifications used in the Zarit Burden Interview. For example, one study reported that most caregivers of individuals with palliative and chronic conditions experience moderate to high levels of burden, with approximately 50.7% reporting severe burden; these levels are consistent with our findings [43]. Another study in a primary healthcare setting identified similarly high levels of severe burden among informal caregivers of individuals with PC needs, underscoring the relevance of this issue beyond specialized PC services [44].

Caregiver burden is increasingly recognized as a multidimensional phenomenon influenced by clinical complexity, caregiving demands, and emotional and organizational factors. The available evidence emphasizes that the needs of families caring for individuals receiving PC are diverse and complex, encompassing emotional, physical, social, spiritual, and financial dimensions, all of which contribute to the overall burden experience [45]. At the individual level, an estimated 10% to 60% of family caregivers experience adverse psychological and physical consequences (including anxiety, depression, grief, and deteriorating physical health), highlighting the substantial emotional impact associated with caregiving in PC settings [46]. Collectively, these findings support the view that informal caregiving in PC entails a considerable risk of physical and emotional strain, particularly when caregivers provide prolonged and intensive care without adequate support or preparation. In this regard, evidence suggests that caregiver training and empowerment interventions can enhance caregiving competencies and reduce burden, reinforcing the importance of structured support programmes in home-based PC [47].

The sociodemographic profile of caregivers in this study, namely a predominance of women with an average age in their sixties, is consistent with the literature. This pattern reflects the well-documented feminization of informal caregiving, whereby women continue to assume primary responsibility for long-term care within families [12,48,49,50]. Previous research suggests that women tend to provide more intensive and sustained care, which may have cumulative effects on their health and well-being over time [51]. Based on our study findings, the higher levels of burden observed among participants with more education may reflect a more comprehensive understanding of the complexities and demands of caregiving, leading to a heightened appraisal of caregiving burden [52].

In bivariate analyses, spouse caregivers reported higher burden than non-spouse caregivers. However, this association was not retained after adjustment for psychosocial and clinical variables. Previous studies have identified spouses as a particularly vulnerable group due to the intensity of the emotional bond, daily cohabitation, and the central role of the marital relationship within the illness experience [38,49,53]. The anticipation of loss, combined with the overlap between spousal and caregiving roles, may intensify the emotional impact of caregiving.

As expected, one of the most important findings of this study was the association between anticipatory grief and caregiver burden. In the multivariable model, higher anticipatory grief scores were independently associated with higher caregiver burden scores. Previous research suggests that emotional responses to progressive losses may intensify caregiving distress and perceptions of burden [54]. This result reinforces the conceptualization of anticipatory grief as a multidimensional emotional response to the progressive losses associated with the deterioration of a loved one [36]. Recent evidence suggests that anticipatory grief encompasses emotional, cognitive, and existential dimensions that can substantially increase psychological distress and perceptions of burden [24].

These findings are consistent with previous research suggesting a complex and potentially bidirectional dynamic between anticipatory grief and caregiver burden, whereby high levels of burden may also exacerbate anticipatory grief [48] and caregiver burden may be important to explain variations in grief experiences [55,56]. Within the context of home-based care, anticipatory grief is particularly salient due to the progressive loss of autonomy and the growing awareness of impending death, contributing to significant emotional distress [57]. Family needs also emerged as a central aspect of the caregiving experience. Although caregivers attributed high importance to care-related needs, they reported lower levels of satisfaction regarding their fulfilment, suggesting gaps in the support available to families. This finding is consistent with the Family Inventory of Needs (FIN) framework which emphasizes the importance of information, communication, and emotional support [36]. Likewise, previous studies have shown that, despite frequently seeking support from healthcare professionals, particularly nurses, caregivers often report unmet needs regarding information and preparation for caregiving [12,53,57,58]. More broadly, families of individuals receiving PC often experience multiple and complex needs across emotional, physical, social, spiritual, and financial domains [45].

Notably, higher needs-satisfaction scores were associated with lower levels of caregiver burden. This finding is consistent with recent evidence indicating that caregivers who feel adequately informed and supported are more likely to adapt successfully to their caregiving role and experience better psychological outcomes [53,59]. In Portugal, recent studies have highlighted that caregivers frequently feel insufficiently prepared for caregiving responsibilities and face structural and organizational limitations in home-care support services, reinforcing the need for more proactive and family-centred care models [60,61].

Regarding patient-related characteristics, caregivers reporting burden also reported higher caregiver-proxy ratings of symptom severity in care recipients (particularly fatigue, appetite loss, anxiety, depression and impaired well-being), reinforcing the influence of patient suffering on the caregiving experience [49,62]. These findings align with evidence indicating that physical and psychological symptoms increase caregiving demands and contribute to greater emotional distress among caregivers [63]. However, when symptom variables were considered simultaneously, caregiver-proxy-rated pain and nausea remained statistically associated with caregiver burden, whereas associations observed for several other symptoms in bivariate analyses were attenuated after adjustment [64]. Unexpectedly, higher caregiver-proxy-rated pain and nausea scores were associated with lower caregiver burden after adjustment. Because these variables were not directly measured, explanations involving greater access to professional support or more effective symptom-management resources should be regarded as speculative hypotheses. Alternative explanations include residual confounding, suppression effects arising from correlations among symptom variables, model-dependent associations, and chance findings, particularly in the context of multiple statistical comparisons. Accordingly, these findings should be interpreted cautiously and require replication in future studies.

Although previous studies have reported that there is a greater caregiver burden among caregivers of individuals with poorer functional status [65], and functional status is a central clinical indicator in PC [34], PPS scores were not significantly associated with caregiver burden after adjustment for psychosocial and clinical variables. The attenuation of the PPS association after adjustment suggests that its relationship with caregiver burden may be partly explained by psychosocial variables and symptom-related characteristics. Further longitudinal research is needed to clarify the relationship between functional decline and caregiver burden.

Overall, the findings reinforce the multidimensional nature of caregiver burden and highlight the interaction between emotional, family-related, and clinical factors. Caregiver burden should therefore be understood as a dynamic and multifactorial phenomenon resulting from the interplay of multiple dimensions of caregiving experience. Informal caregivers frequently face the challenge of balancing caregiving responsibilities with family, occupational, and personal demands, which contributes to elevated levels of stress and burden [66]. Taken together, these results support the importance of integrated strategies that include the early assessment of caregiver burden, the identification of emotional distress, and a structured response to family needs throughout the illness trajectory. Evidence also suggests that caregiver training and empowerment interventions can enhance caregiving skills and reduce burden, further supporting the implementation of structured support programmes within home-based PC services [47].

In sum, caregiver burden was commonly reported in this online sample of family caregivers of individuals perceived to have PC needs. Higher anticipatory grief scores were associated with higher caregiver burden scores, whereas higher family-needs satisfaction scores were associated with lower scores. Given the cross-sectional nature of the study, these findings should be interpreted as associations rather than evidence of causal relationships. Nevertheless, they highlight the importance of routinely assessing caregiver burden, anticipatory grief, and family needs as part of family-centred PC.

### 4.1. Strengths and Study Limitations

This study contributes to the limited Portuguese literature on caregiver burden among family caregivers of individuals perceived to have PC needs. The strength of the study is the simultaneous assessment of caregiver burden, anticipatory grief, family needs, and care recipient characteristics using established instruments with satisfactory psychometric properties. The comparatively large online sample also provides valuable information on the experiences of family caregivers in Portugal and allows the examination of multiple variables associated with caregiver burden.

Nevertheless, several limitations should be considered when interpreting the findings. First, the cross-sectional design does not permit conclusions regarding the temporal ordering or direction of the observed associations. The relationships identified should therefore be interpreted as associations rather than evidence of causal pathways.

Second, eligibility was determined through caregiver self-reports, and PC needs were not verified using a standardized identification framework or clinician confirmation. Consequently, it cannot be assumed that all care recipients met formal criteria for specialist PC referrals, and some degree of population misclassification may have occurred.

Third, the use of convenience and snowball sampling, combined with online recruitment, may have introduced several sources of selection bias. Participation was voluntary, creating the potential for volunteer bias, whereby caregivers with greater interest in the topic, stronger engagement with support services, or higher levels of burden and emotional distress may have been more likely to participate. Conversely, caregivers facing the highest caregiving demands may have been less able to complete the survey. Recruitment through healthcare networks, caregiver organisations, social media platforms, and participant referrals may also have favoured the inclusion of individuals who were socially connected, digitally engaged, or already receiving some form of support. In addition, the exclusive use of an online survey may have excluded caregivers with limited internet access, lower digital literacy, older age, or greater socioeconomic disadvantage. Because recruitment relied partly on open dissemination and snowball sampling, the number of individuals exposed to the study invitation was unknown and a response rate could not be calculated. Network-based recruitment may also have resulted in clusters of participants with similar characteristics. Therefore, the sample cannot be considered representative of Portuguese family caregivers, and the observed proportion of caregiver burden should not be interpreted as a national prevalence estimate but rather as the proportion reported within this self-selected online sample.

Fourth, all care recipient variables, including symptom burden and functional status, were obtained through caregiver-proxy assessments. Although proxy reports are frequently used when direct patient assessment is not feasible, caregiver ratings may differ from patient self-reports, particularly for subjective domains such as depression, anxiety, and well-being. In addition, caregiver burden, anticipatory grief, family-needs satisfaction, symptom burden, and functional status were all reported by the same respondent, creating the possibility of common-method variance and shared-reporting bias.

A further limitation relates to the potential overlap between the assessed constructs. Caregiver burden, anticipatory grief, and family-needs satisfaction were measured using self-report instruments administered at the same time point, and some conceptual overlap may exist between specific domains of these measures, particularly between caregiver burden and the Personal Sacrifice and Burden dimension of the MM-CGI-SF. As a result, associations between these variables may partially reflect shared content and common measurement methods. Alternative explanations, including reciprocal relationships between burden, grief, and perceptions of unmet needs, are also plausible.

Several potentially relevant variables were not collected, including care recipient diagnosis, disease stage, caregiving duration and intensity, caregiver health status, and formal support utilisation. The absence of these variables may have limited a more comprehensive understanding of caregiver experiences. In addition, although burden categories are commonly used in clinical research, categorising continuous ZBI scores may reduce variability and result in some loss of information. Findings based on burden categories should therefore be interpreted alongside analyses using continuous scores.

Future research would benefit from longitudinal designs capable of examining how caregiver burden, anticipatory grief, and family needs are related over time. Studies including more detailed clinical and caregiving information, probabilistic sampling strategies, and multiple data-collection modalities could improve representativeness and strengthen interpretation of findings. Mixed-methods approaches may also contribute to a deeper understanding of the emotional, relational, and contextual dimensions of caregiving in PC.

### 4.2. Implications for Practice

The results of this study demonstrate that caregiver burden was associated not only with the demands of caregiving but also with psychosocial factors, particularly anticipatory grief and the fulfilment of family needs. These results reinforce the importance of a family-centred approach that recognizes family caregivers both as partners in care and as recipients of care themselves [67]. The early identification of caregivers experiencing high levels of anticipatory grief and unmet needs should be a clinical priority, enabling the implementation of interventions aimed at alleviating emotional distress and preventing caregiver burden [68]. In this regard, the systematic assessment of family needs throughout the illness trajectory may contribute to more appropriate care planning and a more personalized response to caregivers’ needs. Home-based and community PC teams play a central role in reducing caregiver burden through the coordination of formal and informal resources, the provision of emotional support, and the promotion of continuity of care [69,70]. In addition, the present findings support the routine assessment of caregiver burden, anticipatory grief, and family needs in community and home-care settings. Although previous research has suggested potential benefits of psychoeducational and support interventions [71,72,73], the current study did not evaluate interventions and therefore cannot determine their effectiveness. Overall, our results support the integration of caregiver burden, anticipatory grief, and family needs assessment into routine clinical practice and highlight the importance of health policies and care strategies that effectively recognize and support family caregivers within the context of home-based care.

## 5. Conclusions

Caregiver burden represents an important concern among family caregivers providing home-based care and warrants attention within family-centred PC practice. In this study, caregiver burden was observed frequently among family caregivers of individuals with perceived PC needs receiving care at home. Caregivers reporting burden also reported higher levels of anticipatory grief, lower satisfaction with family needs, and greater patient symptom burden. In the multivariable analysis, anticipatory grief showed the largest standardized association with caregiver burden, while greater satisfaction with family needs was associated with lower levels of burden. Sex and academic qualifications were also significant factors associated with caregiver burden, with women and caregivers with higher educational attainment reporting higher levels of burden. Furthermore, higher caregiver-proxy-rated levels of pain and nausea among care recipients were independently associated with reduced caregiver burden. These findings highlight the multifaceted nature of caregiver burden and the relevance of considering emotional, family-related, and clinical dimensions when assessing family caregivers in home-based care. The results also support the inclusion of caregiver burden, anticipatory grief, and family needs in routine caregiver assessment and reinforce the importance of family-centred approaches that acknowledge caregivers as both partners in care and individuals with their own support needs.

As this study employed a cross-sectional design and a non-probabilistic sampling strategy, the findings should be interpreted as associations within the study sample rather than evidence of causal relationships or population-level estimates. Furthermore, care recipient clinical characteristics were based on caregiver reports. Future longitudinal studies involving clinically characterized PC populations are needed to further examine these associations and their evolution over the illness trajectory.

## Figures and Tables

**Table 1:** Sample description of caregivers and their care recipients.

**Variables of Caregivers**		**N**	**%**
Sex (n = 401)	Female	327	81.5
	Male	74	18.5
Marital Status (n = 401)	Married/Common-law marriage	252	62.8
	Single	74	18.5
	Divorced/Separated	46	11.5
	Widowed	29	7.2
Academic Qualifications (n = 401)	No qualifications	13	3.2
	Basic education	154	38.4
	Secondary education	102	25.4
	Higher education	132	32.9
Professional situation (n = 401)	No professional activity	188	46.9
	With professional activity	213	53.1
Degree of kinship (n = 401)	Spouse/Partner	78	19.5
	Adult child	152	37.9
	Parent	103	25.7
	Other relative	68	16.9
Caregiver and care recipient cohabitation (n = 401)	Lives in the same residence	254	63.3
	Does not live in the same residence	147	36.7
		Mean	SD
Age (years) (n = 397)		55.93	13.30
Anticipatory grief (MM-CGI-SF) (n = 401)	Global score	56.02	14.22
Family Inventory Needs (FIN)	Importance of Care Needs subscale (n = 397)	93.69	8.95
	Fulfilment of Care Needs subscale (n = 396)	50.12	9.58
Burden Interview (ZBI) (n = 401)	Impact of caregiving	28.46	9.73
	Interpersonal relationship	9.98	3.82
	Expectations regarding care	14.73	3.15
	Sense of self-efficacy	4.71	2.24
**Variables of Care Recipients**		**Mean**	**SD**
Palliative Performance Scale (PPS)	Global score (n = 399)	51.50	15.90
Edmonton Symptom Assessment System-revised (ESAS-r)	Pain (n = 401)	4.67	3.18
	Fatigue (n = 400)	5.83	2.89
	Drowsiness (n = 401)	4.81	3.00
	Nausea (n = 399)	1.92	2.78
	Appetite loss (n = 400)	3.93	3.10
	Shortness of breath/Dyspnoea (n = 401)	2.37	3.00
	Depression (feeling sad) (n = 400)	5.32	3.09
	Anxiety (feeling nervous) (n = 400)	5.09	3.11
	Well-being (n = 400)	5.12	2.63

MM-CGI-SF = Marwit-Meuser Caregiver Grief Inventory-Short Form; FIN = Family Inventory of Needs; ZBI = Zarit Burden Interview; PPS = Palliative Performance Scale; ESAS-r = Edmonton Symptom Assessment System-revised. Higher scores indicate higher anticipatory grief, greater perceived importance and fulfilment of care needs, higher caregiver burden, better functional status (PPS), and greater symptom severity (ESAS-r), respectively.

**Table 2:** Perceived caregiving burden in the caregivers (N = 401).

Caregiver Burden	N	%
No burden (≤45)	92	22.9
Mild burden (46–56)	101	25.2
Severe burden (≥57)	208	51.9

**Table 3:** Bivariate associations according to caregiver burden status (N = 401).

Variables	Categories	Caregivers Burden (ZBI)				Test §	*p*-Value (Two-Tailed)
		No		Yes			
		Nº	%	Nº	%		
Sex	Female	70	76.1	257	83.2	2.364	0.128
	Male	22	23.9	52	16.8		
Marital Status	Married/Common-law marriage	58	63	194	62.8	2.300	0.513
	Single	13	14.1	61	19.7		
	Divorced/Separated	13	14.1	33	10.7		
	Widowed	8	8.7	21	6.8		
Academic Qualifications	No qualifications	4	4.3	9	2.9	4.059	0.255
	Basic education	32	34.8	122	39.5		
	Secondary education	30	32.6	72	23.3		
	Higher education	26	28.3	106	34.3		
Professional situation	No professional activity	39	42.4	149	48.2	0.967	0.325
	With professional activity	53	57.6	160	51.8		
Degree of kinship	Spouse	11	12	67	21.7	4.280	**0.039**
	Non-spouse	81	88	242	78.3		
Caregiver and care recipient cohabitation	Lives in the same residence	53	57.6	201	65	1.690	0.218
	Does not live in the same residence	39	42.4	108	35		

ZBI: Zarit Burden Interview. §: Pearson’s chi-square test. Bold means statistical significance set at *p* < 0.05.

**Table 4:** Study variables of the participating caregivers with and without burden (N = 401).

Variables		ZBI (Caregivers Burden)	N	Mean	SD	Mean Difference (95% CI)	*t* Test	*p*-Value (Two-Tailed)	Effect Size
Age †		Yes	305	55.89	13.58	−0.16 (−3.28 to 2.95)	−0.103	0.918	d = −0.012 (very small)
		No	92	56.05	12.42				
Anticipatory Grief		Yes	309	60.27	11.64	18.50 (15.72 to 21.29)	12.417	**<0.001**	d = 1.553 (large)
		No	92	41.77	12.80				
FIN †	Importance of Care Needs subscale	Yes	306	93.41	9.53	−1.24 (−3.35 to 0.86)	−1.159	0.247	d = −0.138 (small)
		No	91	94.65	6.85				
	Fulfilment of Care Needs subscale	Yes	306	48.56	9.88	−6.66 (−8.83 to −4.49)	−6.031	**<0.001**	d = −0.723 (medium)
		No	90	55.22	6.33				
PPS †		Yes	307	50.55	15.92	−4.12 (−7.82 to −0.42)	−2.190	**0.028**	d = −0.260 (small)
		No	92	54.67	15.50				
ESAS-r	Pain	Yes	309	4.73	3.21	0.24 (−0.51 to 0.98)	0.631	0.529	d = 0.075 (very small)
		No	92	4.49	3.11				
	Fatigue †	Yes	308	6.01	2.83	0.79 (0.12 to 1.47)	2.315	**0.021**	d = 0.275 (small)
		No	92	5.22	3.01				
	Drowsiness	Yes	309	5.01	2.93	0.88 (0.18 to 1.57)	2.473	**0.014**	d = 0.294 (small)
		No	92	4.13	3.14				
	Nausea †	Yes	307	2.05	2.80	0.56 (−0.09 to 1.21)	1.698	0.090	d = 0.202 (small)
		No	92	1.49	2.67				
	Appetite loss †	Yes	308	4.27	3.05	1.47 (0.76 to 2.18)	4.060	**<0.001**	d = 0.482 (small)
		No	92	2.80	2.99				
	Shortness of breath/Dyspnoea	Yes	309	2.44	3.04	0.32 (−0.38 to 1.02)	0.897	0.370	d = 0.107 (very small)
		No	92	2.12	2.89				
	Depression (feeling sad) †	Yes	308	5.56	3.05	1.05 (0.34 to 1.77)	2.888	**0.004**	d = 0.343 (small)
		No	92	4.51	3.09				
	Anxiety (feeling nervous) †	Yes	308	5.38	2.98	1.24 (0.52 to 1.95)	3.381	**<0.001**	d = 0.402 (small)
		No	92	4.14	3.36				
	Well-being †	Yes	308	5.43	2.56	1.34 (0.74 to 1.94)	4.379	**<0.001**	d = 0.520 (medium)
		No	92	4.09	2.64				

SD: Standard deviation. CI: Confidence Interval. FIN: Family Inventory Needs. PPS: Palliative Performance Scale. ESAS-r: Edmonton Symptom Assessment System-revised. †: Variables with missing cases; d: Cohen’s d; Bold means statistical significance set at *p* < 0.05.

**Table 5:** Hierarchical multiple linear regression models predicting caregiver burden (ZBI total score).

Model	R^2^	Adjusted R^2^	ΔR^2^	ΔF	df	*p* for ΔF
Model 1	0.040	0.025	0.040	2.668	6.380	0.015
Model 2	0.530	0.519	0.490	130.939	3.377	<0.001
Model 3	0.564	0.541	0.034	2.853	10.367	0.002

ZBI total score. N = 387. Model 1: age, sex, academic qualifications, professional situation, relationship to care recipient and cohabitation. Model 2 additionally included anticipatory grief, importance of care needs and fulfilment of care needs. Model 3 additionally included PPS and the nine ESAS-r symptoms.

**Table 6:** Final hierarchical multiple linear regression model predicting caregiver burden.

Variables	B	SE	β	95% CI for B	*p*	VIF
Age	−0.044	0.056	−0.038	−0.154; 0.066	0.430	1.939
Sex (Ref. Female)	−3.040	1.386	−0.077	−5.765; −0.315	**0.029**	1.045
Academic qualifications * (Ref. Higher education)	−3.600	1.253	−0.110	−6.064; −1.136	**0.004**	1.225
Professional situation (Ref. No professional activity)	1.294	1.381	0.042	−1.422; 4.010	0.349	1.684
Degree of kinship (Ref. Spouse)	−0.369	1.656	−0.009	−3.625; 2.887	0.824	1.523
Caregiver and care recipient cohabitation (Ref. Lives in the same residence)	−1.735	1.259	−0.054	−4.211; 0.741	0.169	1.303
Anticipatory grief	0.667	0.043	0.622	0.582; 0.752	**<0.001**	1.368
Importance of Care Needs	−0.032	0.063	−0.019	−0.156; 0.092	0.606	1.141
Fulfilment of Care Needs	−0.287	0.063	−0.180	−0.411; −0.163	**<0.001**	1.286
Functional status (PPS)	−0.001	0.040	−0.001	−0.080; 0.078	0.975	1.330
Pain	−0.539	0.203	−0.110	−0.938; −0.140	**0.008**	1.456
Fatigue	0.315	0.246	0.059	−0.169; 0.799	0.201	1.789
Drowsiness	0.413	0.214	0.081	−0.008; 0.834	0.055	1.471
Nausea	−0.608	0.240	−0.109	−1.080; −0.136	**0.012**	1.570
Appetite loss	0.100	0.196	0.020	−0.285; 0.485	0.609	1.309
Shortness of breath/Dyspnoea	−0.316	0.208	−0.062	−0.725; 0.093	0.130	1.386
Depression (feeling sad)	−0.072	0.240	−0.014	−0.544; 0.400	0.764	1.948
Anxiety (feeling nervous)	0.328	0.222	0.067	−0.109; 0.765	0.139	1.723
Well-being	0.240	0.258	0.041	−0.267; 0.747	0.354	1.640

B = unstandardized regression coefficient; SE = standard error; β = standardized coefficient; CI = confidence interval; VIF = variance inflation factor; * variable dichotomized into non-higher education and higher education groups; PPS = Palliative Performance Scale. Bold indicates *p* < 0.05.

## Data Availability

The data that support the findings of this study are available from the corresponding author upon reasonable request. Data are not publicly available because participants did not provide consent for public data sharing and disclosure could compromise confidentiality. The study was not preregistered, and no publicly accessible statistical analysis plan was developed prior to data analysis.

## Supplementary Materials

The following supporting information can be downloaded at https://www.mdpi.com/article/10.3390/jcm15208011/s1, Supplementary file S1: STROBE statement-checklist of items for cross-sectional studies [30] and CHERRIES checklist—checklist for reporting results of internet e-surveys [31]. Supplementary Table S1: Model fit, incremental contribution, and diagnostic statistics for the hierarchical multiple linear regression predicting caregiver burden. Supplementary Table S2: Stability of the main regression coefficients in the sensitivity analysis.

## Abbreviations

The following abbreviations are used in this manuscript:

ESAS-r	Edmonton Symptom Assessment System-revised
FIN	Family Inventory Needs
MM-CGI-SF	Marwit-Meuser Caregiver Grief Inventory-Short Form
PC	Palliative Care
RNCCI	National Network of Integrated Continuous Care
ZBI	Zarit Burden Interview
