# Supplementary material for: Caregiver Burden in an Online Sample of Family Caregivers: Cross-Sectional Associations with Anticipatory Grief and Family Needs
Source: J Clin Med. 2026 Oct 15;15(20):8011. doi: 10.3390/jcm15208011 (PMC13706454; doi:10.3390/jcm15208011)
Supplement: Supplementary file 1 [file jcm-15-08011-s001.zip › Supplementary_Table S2_R3.pdf]

Supplementary Table S2. Stability of the main regression coefficients in the sensitivity analysis

Variables*	β primary (n=387)	p	β sensitivity (n=384)	*p*
Anticipatory grief	0.622	<0.001	0.626	<0.001
Fulfilment of care needs	−0.180	<0.001	−0.186	<0.001
Sex	−0.077	0.029	−0.079	0.024
Academic qualifications	−0.110	0.004	−0.126	<0.001
Pain	−0.110	0.008	−0.115	0.005
Nausea	−0.109	0.012	−0.099	0.019

Note. *Only these variables were included in this table because they were the ones that remained statistically significant in the final model of the primary analysis. Sensitivity model: N = 384; R^2 = 0.581; adjusted R^2 = 0.559; clinical-block ΔR^2 = 0.036; $p < 0.001$. The primary analysis included all 387 complete cases. A sensitivity analysis was conducted after excluding three observations showing the most pronounced residual/influence diagnostics, resulting in an analytical sample of 384 participants. These observations were retained in the primary analysis and were excluded only for the sensitivity analysis to evaluate the robustness of the model estimates. The overall model fit, incremental contribution of the predictor blocks, and direction and statistical significance of the principal regression coefficients remained substantively unchanged.
