# Supplementary material for: Caregiver Burden in an Online Sample of Family Caregivers: Cross-Sectional Associations with Anticipatory Grief and Family Needs
Source: J Clin Med. 2026 Oct 15;15(20):8011. doi: 10.3390/jcm15208011 (PMC13706454; doi:10.3390/jcm15208011)
Supplement: Supplementary file 1 [file jcm-15-08011-s001.zip › jcm-4515905-supplementary_file_S1_R3.pdf]

STROBE STATEMENT - CHECKLIST OF ITEMS FOR CROSS-SECTIONAL STUDIES [30]

Manuscript: Caregiver Burden in an Online Sample of Family Caregivers: Cross-Sectional Associations with Anticipatory Grief and Family Needs

Section/Topic	Item #	Recommendation	Reported on page/section #
Title and abstract	1	(a) Indicate the study's design with a commonly used term in the title or the abstract. (b) Provide an informative and balanced summary of what was done and what was found.	p.1, Title; p.1, Abstract
Introduction — Background/rationale	2	Explain the scientific background and rationale for the investigation being reported.	p.2-3, Introduction
Introduction — Objectives	3	State specific objectives, including any prespecified hypotheses.	p.2, Introduction
Methods — Study design	4	Present key elements of study design early in the paper.	p.3, Study Design
Methods — Setting	5	Describe the setting, locations, and relevant dates, including periods of recruitment, exposure, follow-up, and data collection.	p.3, Study Setting; p.5-6, Data Collection
Methods — Participants	6	(a) Give the eligibility criteria, and the sources and methods of selection of participants.	p.4, Participants and recruitment
Methods — Variables	7	Clearly define all outcomes, exposures, predictors, potential confounders, and effect modifiers. Give diagnostic criteria, if applicable.	p.4, Instruments
Methods — Data sources/measurement	8	For each variable of interest, give sources of data and details of methods of assessment (measurement). Describe comparability of assessment methods if more than one group.	p.4-5, Instruments
Methods — Bias	9	Describe any efforts to address potential sources of bias.	p.6, Data Collection; p.6, Data Analysis
Methods — Study size	10	Explain how the study size was arrived at.	p.4, Participants and recruitment
Methods — Quantitative variables	11	Explain how quantitative variables were handled in the analyses. If applicable, describe which groupings were chosen and why.	p.6, Data Analysis
Methods — Statistical methods	12	(a) Describe all statistical methods, including those used to control for confounding. (b) Describe any methods used to examine subgroups and interactions. (c) Explain how missing data were addressed. (d) If applicable, describe analytical methods taking account of sampling strategy. (e) Describe any sensitivity analyses.	p.6-7, Data Analysis

Section/Topic	Item #	Recommendation	Reported on page/section #
Results — Participants	13	(a) Report numbers of individuals at each stage of study — eg numbers potentially eligible, examined for eligibility, confirmed eligible, included in the study, completing follow-up, and analysed. (b) Give reasons for non-participation at each stage. (c) Consider use of a flow diagram.	p.4, Participants and recruitment
Results — Descriptive data	14	(a) Give characteristics of study participants (eg demographic, clinical, social) and information on exposures and potential confounders. (b) Indicate number of participants with missing data for each variable of interest.	p.7-8, Results; Table 1
Results — Outcome data	15	Report numbers of outcome events or summary measures.	p.8–11, Results; Tables 2–5
Results — Main results	16	(a) Give unadjusted estimates and, if applicable, confounder-adjusted estimates and their precision (eg 95% confidence interval). Make clear which confounders were adjusted for and why they were included. (b) Report category boundaries when continuous variables were categorized. (c) If relevant, consider translating estimates of relative risk into absolute risk for a meaningful time period.	p.8–11, Inferential Analysis; Tables 3–5
Results — Other analyses	17	Report other analyses done — eg analyses of subgroups and interactions, and sensitivity analyses.	p.12, Results; Supplementary Table S2
Discussion — Key results	18	Summarise key results with reference to study objectives.	p.11, Discussion (opening)
Discussion — Limitations	19	Discuss limitations of the study, taking into account sources of potential bias or imprecision. Discuss both direction and magnitude of any potential bias.	p.14–15, Strengths and Study Limitations
Discussion — Interpretation	20	Give a cautious overall interpretation of results considering objectives, limitations, multiplicity of analyses, results from similar studies, and other relevant evidence.	p.11–13, Discussion
Discussion — Generalisability	21	Discuss the generalisability (external validity) of the study results.	p.14-15, Strengths and Limitations
Other information — Funding	22	Give the source of funding and the role of the funders for the present study and, if applicable, for the original study on which the present article is based.	p.16, Declarations — Funding

Note: An explanation and elaboration article discusses each checklist item and gives methodological background and published examples of transparent reporting. The STROBE checklist is best used in conjunction with this article (freely available on the websites of PLoS Medicine at http://www.plosmedicine.org/, Annals of Internal Medicine at http://www.annals.org/, and Epidemiology at http://www.epidem.com/). Information on the STROBE Initiative is available at http://www.strobe-statement.org.

CHERRIES CHECKLIST — CHECKLIST FOR REPORTING RESULTS OF INTERNET E-SURVEYS [31]

Manuscript: Caregiver Burden in an Online Sample of Family Caregivers: Cross-Sectional Associations with Anticipatory Grief and Family Needs

Item Category	Item #	Checklist Item	Reported on page/section #
Design	1	Describe survey design (eg cross-sectional, longitudinal, panel).	p.3, Study Design — cross-sectional design clearly stated
IRB approval and informed consent	2	(a) Mention whether the study has been approved by an IRB. (b) Describe consent process.	p.7, Ethical Considerations
Data protection	3	If any personal information was collected or any...identifiable data, describe what mechanisms were used to protect data.	p.5-6, Data Collection; p.7 Ethical considerations
Development and pre-testing	4	State how the survey was developed, including whether it was pre-tested.	p.4-5, Instruments; No formal pilot testing or pre-testing of the assembled online questionnaire was conducted before study implementation.
Recruitment process and description of the sample	5	Describe the process of recruitment, eg invitation by email or web page; survey method (eg internet, mail).	p.5-6, Data Collection — distribution channels described
Survey administration	6	(a) State the type of e-survey (eg distributed via mailing list or web/online survey). (b) Describe survey context (e.g. website look, advertisement). (c) Mention if the survey was incentivised.	p.5-6, Data Collection — Google Forms platform described
Response rates	7	(a) Calculate response rate (number completed/number invited). (b) Calculate completion rate.	p.4, Participants and recruitment; A conventional response rate was not calculated because the number of individuals exposed to the open invitation was unknown. However, among the 427 individuals who initiated the survey, 401 completed all items of the primary outcome measures and were included in the analysis, corresponding to a completion rate of 93.9%.

Item Category	Item #	Checklist Item	Reported on page/section #
Preventing multiple entries from the same individual	8	Describe steps taken to prevent multiple entries from the same individual.	p.6, Data Collection — instructional duplicate prevention described; no technical methods (e.g., cookies, IP tracking, registration systems) for duplicate-response prevention were available.
Analysis	9	(a) Describe handling of incomplete questionnaires. (b) Describe statistical correction techniques.	p.6-7, Data Analysis; No formal correction for multiple comparisons was applied.
