# Supplementary material for: Caregiver Burden in an Online Sample of Family Caregivers: Cross-Sectional Associations with Anticipatory Grief and Family Needs
Source: J Clin Med. 2026 Oct 15;15(20):8011. doi: 10.3390/jcm15208011 (PMC13706454; doi:10.3390/jcm15208011)
Supplement: Supplementary file 1 [file jcm-15-08011-s001.zip › Supplementary_Table S1_R3.pdf]

Supplementary Table S1. Model fit, incremental contribution, and diagnostic statistics for the hierarchical multiple linear regression predicting caregiver burden

Domain	Indicator	Model 1	Model 2	Model 3 / Final model	Interpretation
Analytical sample	N	387	387	387	Same complete-case sample across all models
Model composition	Variables added	Sociodemographic and caregiving characteristics	+ Anticipatory grief and family-needs variables	+ Functional status and caregiver-proxy-rated symptoms	Predictors entered cumulatively
Model fit	R	0.201	0.728	0.751	Increasing model-outcome association
	R^2	0.040	0.530	0.564	Final model explained 56.4% of ZBI variance
	Adjusted R^2	0.025	0.519	0.541	54.1% after adjustment for number of predictors
	Standard error of estimate	15.215	10.690	10.436	Prediction error decreased across models
Incremental contribution	ΔR^2	0.040	0.490	0.034	Blocks contributed 4.0%, 49.0%, and 3.4%, respectively
	ΔF	2.668	130.939	2.853	Significant improvement at each step
	df for ΔF	6.380	3.377	10.367	Degrees of freedom for F-change
	p for ΔF	0.015	<0.001	0.002	Each block significantly improved explained variance
Overall model ANOVA	F	2.668	47.249	24.983	All three cumulative models statistically significant
	df	6.380	9.377	19.367	
	p	0.015	<0.001	<0.001	
Multicollinearity, final model	Tolerance, range			0.513 to 0.957	No evidence of problematic multicollinearity
	VIF, range			1.045 to 1.948	No evidence of problematic multicollinearity
Residual independence	Durbin-Watson			1.596	No substantial residual autocorrelation indicated
Residual diagnostics	Standardized residuals, range			−3.100 to 4.404	Some relatively large residuals present
	Studentized residuals, range			−3.137 to 4.519	A small number of potentially atypical observations
	Residual mean			0.000	Residuals centered around zero
	Residual SD			10.176	Dispersion of raw residuals
Influence diagnostics	Cook's distance, maximum			0.061	No observation showed substantial overall influence

	Mahalanobis distance, maximum			81.272	Indicates observations with relatively unusual predictor profiles; considered alongside influence measures
	Centered leverage, maximum			0.211	Some high-leverage observations present
Predictive/model information	PRESS			44,511.271	Prediction-error diagnostic
	AIC	2113.964	1843.681	1834.710	Lower values indicate improved relative fit, although AIC should be interpreted comparatively
	BIC/Schwarz criterion	2141.673	1883.265	1913.878	Model 2 has the lowest BIC among the three models

Note. Outcome variable: total Zarit Burden Interview (ZBI) score. Model 1 included age, sex, educational level, professional status, degree of kinship, and caregiver-care recipient cohabitation. Model 2 additionally included anticipatory grief, importance of care needs, and fulfilment of care needs. Model 3 additionally included functional status (PPS) and the nine ESAS-r symptoms. Predictors were entered cumulatively, and all 19 predictors were retained in the final model. Complete-case analysis was used. ΔR^2 = change in coefficient of determination; ΔF = F test for change in R^2; VIF = variance inflation factor; PRESS = predicted residual error sum of squares; AIC = Akaike information criterion; BIC = Bayesian information criterion; PPS = Palliative Performance Scale; ESAS-r = Edmonton Symptom Assessment System-revised. Visual inspection of the standardized residual histogram, normal probability plot, and plot of standardized residuals against standardized predicted values indicated no major departures from approximate normality of residuals, linearity, or homoscedasticity.
